# Does the New Rural Cooperative Medical Scheme Provincial Pooling Policy Improve Health Equity Among Older Adults? – Evidence From China Longitudinal Healthy Longevity Survey Data

**DOI:** 10.34172/ijhpm.8671

**Published:** 2025-05-21

**Authors:** Miao Peng, Dai Baozhen, Liao Xin

**Affiliations:** Department of Labor and Social Security, School of Public Health, Southeast University, Nanjing, China.

**Keywords:** NRCMS, Provincial Pooling, Older Adults, Health, Health Inequalitie

## Abstract

**Background:**

As global aging increases, health inequalities are becoming more prominent. The purpose of this study is to examine whether increasing the level of fund pooling of the New Rural Cooperative Medical Scheme (NRCMS) helps to improve health and health inequalities among older adults.

**Methods:**

Data from four periods of the China Longitudinal Healthy Longevity Survey (CLHLS) in 2008, 2011, 2014, and 2018 were used, the population for this paper was a sample of the older adults participating in the NRCMS. A sample of 955 treated participants and 13 477 control participants were included in the analysis after excluding samples with missing information. Time-varying difference-in-differences (DID) model was used to analyze the impact of the NRCMS Provincial Pooling Policy (NRCMS-PPP) on participants’ health and health inequalities.

**Results:**

The results of the study showed that the NRCMS-PPP had a significant effect on the self-rated health (SH) (estimated coefficient = 0.149, P<.01) and health relative deprivation index (HRDI) (estimated coefficient = -0.018, *P*=.02). Further exploration of the heterogeneous effect of it revealed that implementation is more effective in improving the health and reducing health inequalities for older population with primary education or living in rural areas. The mediation mechanism suggests that NRCMS-PPP partially mediates through total out-of-pocket medical expenses (TOME) and catastrophic health expenditure (CHE).

**Conclusion:**

The NRCMS-PPP reduces the probability of the older adults experiencing CHE and reduces their burden of disease costs, thus improving their health and reducing their health inequality. Policy effects vary in terms of educational status and areas of residence.

## Introduction

**Key Messages**

**Implications for policy makers**

- First, this study will help to continue to promote the New Rural Cooperative Medical Scheme Provincial Pooling Policy (NRCMS-PPP), continuously raise its level of fund pooling, and ultimately achieve national fund pooling.
- Second, the results will prompt policy-makers to choose the mode of provincial integration according to the actual situation. For regions with a low level of economic development or large differences in economic levels within the region, the Risk Transfer Fund Model (RTFM) can be chosen first, and then gradually transitioned to the Unified Revenue and Expenditure Model (UREM) as the level of economic development and inter-regional differences are reduced.
- Third, this study will remind policy-makers to scientifically formulating incentive and supervision mechanisms, strengthening the supervision of medical insurance funds, and doing a good job of hierarchical diagnosis and treatment, so as to guide insured persons to make rational use of medical resources.

**Implications for the public**

This study will help to promote the provincial pooling of basic medical insurance and raise its level of fund pooling. This will make it easier for people to seek medical treatment in other places and enhance their accessibility to medical care. In addition, the implementation of this policy will allow participating farmers in the province to enjoy the same reimbursement and compensation for medical treatment, thus improving the level of medical protection in areas with a lower level of economic development, improving people’s health, and alleviating health inequality. However, while raising the level of fund pooling, it is necessary to strengthen the supervision of the health insurance fund to avoid moral hazard and excessive medical treatment arising from the increase in the level of medical insurance benefits.

Against the backdrop of a growing global economy, people’s health continues to improve and life expectancy continues to increase. But at the same time, persistent health inequalities have come to the fore and can be observed between the following categorized social groups,^1,2^ which can include socioeconomic status, gender and ethnicity.^3-5^ The issues of health inequalities affect every individual in society and have received widespread attention. By the end of 2022, Chinese population aged 60 years and above was 280.04 million, accounting for 19.8% of the total population.^6^ According to data from the National Health Commission, nearly 80% of the 210 million older adults over 65 will suffer from at least one chronic disease by the end of 2022, making the problem of “longevity and ill-health” among older population more prominent.^7^ Kawachi et al found that the health of older persons is influenced by a number of factors, including income, lifestyle, accessibility of medical services, and social status.^8-10^ Disparities in income, health-care accessibility and social status exacerbate health inequalities by further stretching the health of the older population.^11^

 Health inequalities have attracted much attention and widespread discussion.^12,13^ The “Healthy China 2030” introduces the concept of health equality into China’s aging field and prioritizes health as a development strategy.^14^ However, due to differences in the level of economic development between urban and rural areas, there are large disparities in the utilization of healthcare services among different residents, thus exacerbating health inequalities. Health inequalities do not refer to health disparities in the general sense, but rather to systematic health disparities triggered by having different endowments.^15^ Data from China’s seventh population census in 2020 showed that about 16.10% of people aged 60 or older living in rural areas were unhealthy. This compares with about 8.14% in urban areas,^16^ and health inequalities among older persons in rural areas are particularly prominent in China.^17,18^ The lower level of pooling of the New Rural Cooperative Medical Scheme (NRCMS) is difficult to effectively utilize the fund’s co-payment capacity, which is not conducive to the improvement of health equity. TheNRCMS has made remarkable achievements since its implementation in 2003, significantly improving the health of participants, alleviating the situation of “have no medical care” and reducing their burden of disease costs.^19,20^ The NRCMS has greatly guaranteed the right of farmers to access basic healthcare services. However, there are still some problems with the NRCMS. The low level of health insurance fund pooling has exacerbated the paradox of unbalanced healthcare utilization and unequal healthcare benefits. Therefore, it is urgent to raise the level of fund pooling.

 Prior to the implementation of NRCMS Provincial Pooling Policy (NRCMS-PPP), the province’s health insurance fund pool consisted of decentralized fund pools in various cities and counties (districts), with different NRCMS financing standards and treatment and compensation levels in different cities and counties (districts), and without support for settlement of health insurance for medical treatment in other places. After the implementation of NRCMS-PPP, the province has implemented unified financing standards, compensation policies, business processes, service supervision, and information management to facilitate cross-location medical treatment. According to the management of NRCMS-PPP, it can be categorized into two models: the “Unified Revenue and Expenditure Model (UREM)” and the “Risk Transfer Fund Model (RTFM).” The UREM refers to the consolidation of the province’s NRCMS fund pools into one fund pool with unified management and separate accounting. The RTFM refers to the creation of a pool of funds called Provincial Transfer Funds, which is generally about 5% to 10% of the previous year’s total fund revenue, to address the shortfalls in income and expenditure of some of the fund pool in the province. In fact, the UREM utilizes the principle of the law of large numbers to enhance the risk-resistance and financing capacity of the health insurance fund pools, while the RTFM establishes a reserve fund pool for the normal operation of each fund pool. Both models enhance the fairness of the NRCMS system, improve the co-payment capacity of the NRCMS fund, and facilitate the settlement of settlements for people seeking medical treatment in other places, enhancing the accessibility of medical services. In order to alleviate health inequalities of Chinese rural residents, the Opinions of the State Council of the Central Committee of the Communist Party of China on Deepening the Reform of the Medical and Health Care System proposes to “gradually raise the pooling level of basic medical insurance, and narrow the gap in the level of protection.” As of September 2023, six provinces in China have implemented the NRCMS-PPP. Moreover, Beijing and Tibet further became the Basic Medical Insurance for Urban and Rural ResidentsProvincial Pooling Policy (BMIURR-PPP) by merging the NRCMS and the Basic Medical Insurance for (Non-working) Urban Residents into the BMIURR while completing the NRCMS-PPP.

 Currently, scholars have focused more on the health impacts of the health insurance provincial pooling policy and less on its impact on health inequalities. Bazyar finds that fragmented pools will lead to inefficiencies in the healthcare system, do not fully utilize the pooling capacity of the healthcare funds, and result in inequitable benefits among different populations.^21^ By reviewing and comparing the experiences of merging health insurance fund pools in South Korea, Turkey, Thailand, and Indonesia, Bazyar finds that this has helped to improve equity in access to and utilization of healthcare services for different populations, but there is also a risk of fraud and corruption.^22^ Shen finds that promoting the pooling level of the Basic Medical Insurance for Urban Workers from municipal level to provincial level significantly improves individual health service utilization but does not result in health benefits.^23^ Dong finds that the provincial pooling policy reduces the burden of healthcare costs on participants, and that there is heterogeneity across income and age groups.^24^ Wu et al find that the provincial pooling policy helped to improve the probability of chronic diseases among low-income groups and alleviated the health inequalities associated with income disparities.^25^ In addition, increasing the level of pooling may also have negative effects. Fu et al find that the unclear division of responsibilities among all levels of government after raising the level of pooling could easily lead to the problem of moral hazard, which may induce unnecessary medical behaviors of the insured.^26^ Such unnecessary medical behavior greatly increases medical expenses and is not conducive to the sustainable development of the health insurance fund.^27^ In summary, the provincial pooling policy may have improved health status and health inequalities by improving healthcare coverage in some areas, reducing the burden of healthcare costs, and promoting patient utilization of healthcare services. The innovation of this study is that it focuses on the NRCMS-PPP for the first time, and selects China Longitudinal Healthy Longevity Survey (CLHLS) four-period panel data to empirically explore the impact of NRCMS-PPP on health and health inequality of the older population using the time-varying difference-in-differences (DID) model.

## Methods

###  Data Source

 The data used in this study is sourced from the CLHLS. The questionnaire covered various aspects such as the basic conditions of the older adults and their families, economic status, health status, lifestyle, disease treatment, and medical expenditure burdens. The CLHLS data exhibit good reliability and validity, characterized by comprehensive survey content, a scientifically structured design, large sample size, long tracking periods, and strong representativeness. The research findings based on CLHLS data have been used in peer reviewed studies both domestically and internationally.^28-30^

**Figure 1:**
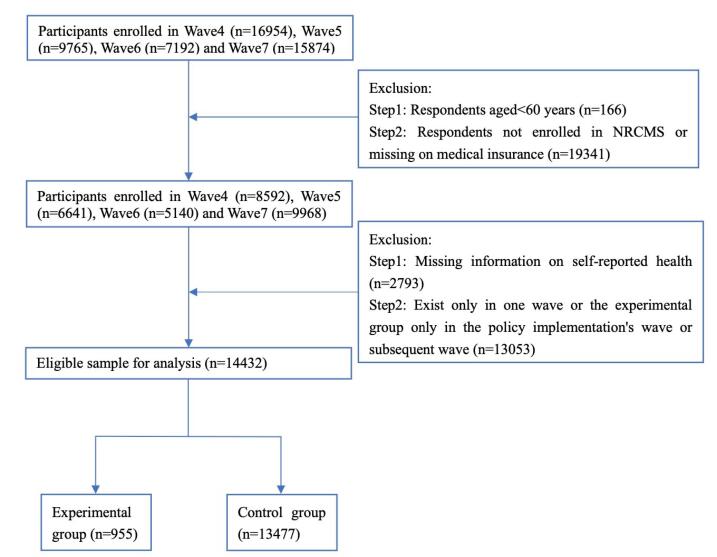
Sample Selection Process Diagram. Abbreviation: NRCMS, New Rural Cooperative Medical Scheme.

 This study utilized panel data from the CLHLS for the years 2008, 2011, 2014, and 2018. Considering the definition of older adults in China, participants aged 60 and above were categorized as older adults. Cleaning data is divided into the following steps: samples with actual ages below 60 were excluded; only samples from participants enrolled in the NRCMS were retained; and samples lacking essential information were removed^[1]^. Ultimately, this study obtained a total of 14 432 valid samples (Figure 1).

###  Statistical Analysis

 Of the 23 provinces included in the CLHLS, five have implemented NRCMS-PPP: Tianjin, Chongqing, Hainan, Shanghai, and Beijing began implementing NRCMS-PPP on January 1, 2010, October 24, 2011, January 1, 2013, January 1, 2015, and January 1, 2018, respectively. Given that the implementation timing of the NRCMS-PPP varies across provinces in China, this research employs a Time-varying DID model to assess the health effects of the NRCMS-PPP on the older population. The 2008-2018 CLHLS waves cover the time before and after the implementation of the NRCMS-PPP well enough to be able to measure the effects of its policies using the time-varying DID model.

 The model is set as follows:


(1) $$Y_{iyp}=\beta_{0}+\beta_{1}Policy_{iyp}+\delta X_{iyp}+\gamma_{i}+\mu_{y}+\delta_{p}+\varepsilon_{iyp}$$

Where *i* (*i* = 1, 2,..., n) represents individuals, and *y* (*y* = 2008, 2011, 2014, 2018) represents the year, and *p* (*p* = Beijing, Jiangsu, …, Hainan) represents the province. *Y*_iyp_ is the dependent variable, representing individual *i*’sself-rated health (SH)and health relative deprivation index (HRDI) in year *y*. *Policy*_iyp_ is the key explanatory variable, indicating whether individual *i*’sregion has implemented the NRCMS-PPP. If implemented, *Policy*_iyp_ = 1; otherwise, it is 0. *X*_iyp_ represents the control variables. *γ*_i_ is individual fixed effect. *µ*_y_ is year fixed effect. *δ*_p_ is province fixed effect. *ɛ*_iyp_ is the random error term.

###  Variable Design

####  Dependent Variable

 The dependent variables in this study are health and health inequalities. The World Health Organization (WHO) defines health as a multidimensional composite indicator. Among various health assessment indicators, SH is widely adopted by many scholars because it comprehensively reflects an individual’s health condition in various aspects, including physiological, psychological, and behavioral factors.^31,32^ As for health inequalities, this study employs the HRDI derived from individual SH measures to assess health inequalities. Referring to previous studies, this research uses the HRDI based on individual SH measures to characterize health inequalities.^33,34^

 According to the theory of Relative Deprivation,^35^ within a community, as residents’ health levels decrease, their health disadvantages increase, leading to a higher degree of health relative deprivation. Based on Kakwani’s definition of the HRDI,^36^ assuming *Y* is a reference group with a sample size of n, the SH levels of people are ranked to obtain the overall distribution vector *Y* = (*y*_1_*, y*_2_*, y*_3_*, …, y*_n-1_*, y*_n_), where *y*_1_ ≤ *y*_2_ ≤ *y*_3_ … ≤ *y*_n-1_ ≤ *y*_n_. According to the definition, comparing the *i* resident *y*_i_ with the *j* resident *y*_j_, the HRDI *RD*(*y*, *y*_i_) for the *i* resident can be expressed as:


(2) $$RD\left(y_{j},y_{i}\right)=\left\{\begin{array}{l} y_{j}-y_{i}\;if\;\left(y_{j}>y_{i}\right)\\ \;0\;\;if\;\left(y_{j}\leq y_{i}\right)\; \end{array}\right.$$

Building upon equation (2), the average Relative Deprivation *RD*(*y*, *y*_i_) experienced by the resident *y*_i_ can be expressed as:


(3) $$RD\left(y,y_{i}\right)=\frac{1}{n\mu_{Y}}\left(n_{y_{i}}^{+}\times \mu_{y_{i}}^{+}-n_{y_{i}}^{+}\times y_{i}\right)$$

*µ*_Y_ is the mean of SH for all samples in the group *Y,* $n_{y_{i}}^{+}$ is the count of samples in group *Y* with SH exceeding *y*_i_, and $n_{y_{i}}^{+}$ is the mean of SH for those samples in group *Y* with SH exceeding *y*_i_.

####  Independent Variable

 “Provincial Pooling” is the explanatory variable in this study, which indicating whether the NRCMS-PPP has been implemented. A value of Provincial Pooling = 1 represents that the province has implemented the NRCMS-PPP, while a value of Provincial Pooling = 0 indicates that the province has not yet implemented the NRCMS-PPP.

####  Other Control Variable

 Referring to Grossman health capital theory model,^37^ control variables were selected from the following five levels: personal characteristics, lifestyle habits, household hygiene environment, accessibility of medical services, and regional economic factors. Personal characteristics include age, gender, education, income, etc. Lifestyle habits encompass smoking, drinking, exercising, etc. The household hygiene environment is measured by indicators such as the water source and musty of house. The accessibility of medical services is indicated by the ability to seek timely medical attention when seriously ill. Regional economic differences are represented by urban-rural disparities.

 As can be seen from Table 1, in the 2008-2018 CLHLS data, the mean value of SH was 3.397, indicating that the majority of the samples were in fair or above fair health. The mean value of the HRDI was 0.131. The mean value of the age was about 83; 46.8% of the samples were male, with a relatively balanced ratio of men to women; 42.4% of the samples had spouses.

**Table 1:** Sample Variable Definition and Descriptive Statistics

**Variable**	**Definition**	**Mean**	**SD**
Dependent variables			
SH	Very bad = 1; bad = 2; fair = 3; good = 4; very good = 5	3.397	0.894
HRDI	0~1, with higher scores indicating greater relative health deprivation among older people	0.131	0.128
Core independent variable			
Provincial pooling	NRCMS-PPP = 1; otherwise = 0	0.031	0.174
Other control variables			
Age	Age of residents (years)	82.847	10.346
Gender	Female = 0; male = 1	0.468	0.499
Co-residence			
With family	Yes = 1, no = 0	0.798	0.402
Alone	Yes = 1, no = 0	0.187	0.390
In a nursing home	Yes = 1, no = 0	0.015	0.120
Education	Illiteracy = 1; primary = 2; middle school and above = 3	1.562	0.740
Family income^a^	Logarithmic treatment of annual household income (CNY)	20 352.080	24 840.730
Smoking	Smoking recently = 1; otherwise = 0	0.201	0.401
Drinking	Drinking recently = 1; otherwise = 0	0.186	0.389
Exercising	Exercising recently = 1; otherwise = 0	0.261	0.439
Water quality	Drinking tap or purified water = 1; drinking wells water, rivers or lakes water, springs or ponds water = 0	0.581	0.493
Musty of house	The house always smells musty = 1; otherwise = 0	0.193	0.395
Medical accessibility	Timely access to medical care in the case of serious illness; otherwise = 0	0.949	0.219
Residency			
City	Yes = 1, no = 0	0.029	0.167
Town	Yes = 1, no = 0	0.286	0.452
Rural	Yes = 1, no = 0	0.685	0.464

Abbreviations: SD, standard deviation; SH, self-rated health; HRDI, health relative deprivation index; NRCMS-PPP, New Rural Cooperative Medical Scheme Provincial Pooling Policy. Note: *a* family income is reported as the statistical result of the original value of the variable.

## Results

###  The Impact of NRCMS-PPP on Health and Health Inequalities

Table 2 shows that the impact of the NRCMS-PPP on the SH and HRDI of the older population. Considering the implementation of BMIURR-PPP in Beijing, the sample in Beijing was excluded to avoid the interference of the integrated urban-rural medical insurance^[2]^. The results indicate that, with or without controlling for variables, NRCMS-PPP has a significant positive impact on the SH of the older population (estimated coefficients = 0.139 and 0.142, respectively; *P* <.01, respectively). NRCMS-PPP has a significant negative impact on the HRDI of the older adults (estimated coefficients are -0.018 and -0.018, respectively; *P* = .03, respectively). These findings suggest that the NRCMS-PPP improves the SH of the older population, reduces their HRDI.

**Table 2:** Impact of NRCMS-PPP on Health and Health Inequalities

**Variables**	**SH**		**HRDI**	
	** (1)**	** (2)**	** (3)**	** (4)**
Provincial pooling	0.139^***^ (2.661)	0.142^***^ (2.710)	-0.018^**^ (-2.149)	-0.018^**^ (-2.166)
Age		0.007 (1.058)		-0.001 (-0.939)
Gender		-0.030 (-0.116)		0.006 (0.213)
Education		0.025 (0.654)		-0.001 (-0.153)
Marital		0.002 (0.042)		0.003 (0.661)
Co-residence				
Alone		0.059^**^ (1.974)		-0.006 (-1.303)
In a nursing home		-0.108 (-1.452)		0.010 (0.952)
Family income		0.030^***^ (4.360)		-0.004^***^ (-3.683)
Smoking		0.039 (1.113)		-0.003 (-0.501)
Drinking		0.078^**^ (2.559)		-0.012^***^ (-2.789)
Exercising		0.130^***^ (6.215)		-0.015^***^ (-4.984)
Water quality		0.035 (1.521)		-0.004 (-1.121)
Musty of house		-0.106^***^ (-3.886)		0.016^***^ (3.851)
Medical accessibility		0.323^***^ (7.484)		-0.052^***^ (-7.578)
Residency				
Town		-0.0143 (-0.236)		0.001 (1.171)
Rural		-0.019 (-0.316)		0.001 (0.081)
Constant	3.391^***^ (2102.162)	2.120^***^ (3.387)	0.132^***^ (525.871)	0.300^***^ (3.261)
Individual FE	√	√	√	√
Year FE	√	√	√	√
Province FE	√	√	√	√
Sample size	14 411	14 411	14 411	14 411
R^2^	0.232	0.246	0.236	0.250

Abbreviations: SH, self-rated health; HRDI, health relative deprivation index; NRCMS-PPP, New Rural Cooperative Medical Scheme Provincial Pooling Policy; FE, fixed-effects. *** and ** represent significance at the 1% and 5% level.

###  Heterogeneity Test Results 

 To further verify the heterogeneity of the implementation effect of NRCMS-PPP, this analysis is carried out in two dimensions: Education and living area. The heterogeneous effects of different educational status were first analyzed. Panel A in Table 3 shows the impact of the NRCMS-PPP on the SH and HRDI, primary, and middle school and above older adults. Regarding health, the policy has a positive effect on older population which are illiterate or with the level of primary education at a significance level of 5%, but the effect is not significant for older adults with the level of middle school education and above. The estimated coefficient is higher for older adults with the level of primary school education than illiterate older adults (Significant difference in subgroup sample coefficients: -0.108, *P* = .02). This indicates that the NRCMS-PPP is more effective in improving the health of older adults with the level of primary school education. In terms of health inequalities, the impact of the policy varied across participants with different levels of education, but was only significant for illiterate older adults (estimated coefficients = -0.018; *P* = .08). This shows that the policy is conducive to reducing the HRDI of the illiterate older adults.

**Table 3:** Analysis of Heterogeneity in NRCMS-PPP

**Panel A: Grouped by Level of Education**		
**Variables **	**SH**	**HRDI**
Illiterate		
Provincial pooling	0.145^**^ (2.204)	-0.018^*^ (-1.720)
Control variables	√	√
Constant	0.738 (0.708)	0.496^***^ (3.086)
Ind, Year, Prov	√	√
Sample size	8493	8493
R^2^	0.233	0.231
Primary		
Provincial pooling	0.253^**^ (2.132)	-0.024 (-1.412)
Control variables	√	√
Constant	5.396^***^ (5.4322)	-0.124 (-0.742)
Ind, Year, Prov	√	√
Sample size	3768	3768
R^2^	0.248	0.251
Middle school and above		
Provincial pooling	0.022 (0.181)	-0.008 (-0.421)
Control variables	√	√
Constant	1.833 (1.061)	0.405 (1.424)
Ind, Year, Prov	√	√
Sample size	2171	2171
R^2^	0.262	0.288
Fisher combined tests	Illiterate - Primary	
	-0.108^**^, *P* =.02	
Sample size	8258	3518
R^2^	0.539	0.546
**Panel B: Grouped by Urban and Rural Areas**		
**Variables**	**SH**	**HRDI**
Town		
Provincial pooling	0.081 (0.531)	-0.001 (-0.031)
Control variables	√	√
Constant	2.422 (1.577)	0.233 (0.863)
Ind, Year, Prov	√	√
Sample size	4124	4124
R^2^	0.261	0.256
Rural		
Provincial pooling	0.137^**^ (2.223)	-0.017^*^ (-1.692)
Control variables	√	√
Constant	2.488^***^ (2.955)	0.303^**^ (2.355)
Ind, Year, Prov	√	√
Sample size	9893	9893
R^2^	0.234	0.229

Abbreviations: SH, self-rated health; HRDI, health relative deprivation index; NRCMS-PPP, New Rural Cooperative Medical Scheme Provincial Pooling Policy. ***, ** and * represent significance at the 1%, 5%, and 10% level.

 The heterogeneous effects of geographic residence were secondly analyzed. Panel B shows the impact of NRCMS-PPP on the SH and HRDI of older adults living in towns and rural areas. The sample size for living in the city was removed because it was too small to analyze (N = 23 treated). For older adults living in towns, the estimated coefficients of the policy on their SH and HRDI are 0.081 and -0.001, respectively, but are not statistically significant. For older adults living in rural areas, the estimated coefficients of the policy on their SH and HRDI are 0.137 and -0.017 with significance levels of 5% and 10%, respectively.

###  Different NRCMS Provincial Pooling Models

 This study analyzes and compares the effects of the NRCMS-PPP on health and health inequalities of older population under different provincial pooling models. Both the UREM and the RTFM have a positive impact on the health status of older population, both significant at the 5% significance level, with estimated coefficients of -0.447 and -0.130, respectively. In terms of health inequalities, only the RTFM has a negative impact on the HRDI with an impact coefficient of -0.017, which is significant at the 5% significance level. Please see appendix for specific results

###  Results of the Robustness Test

####  Parallel Trend Test

**Figure 2:**
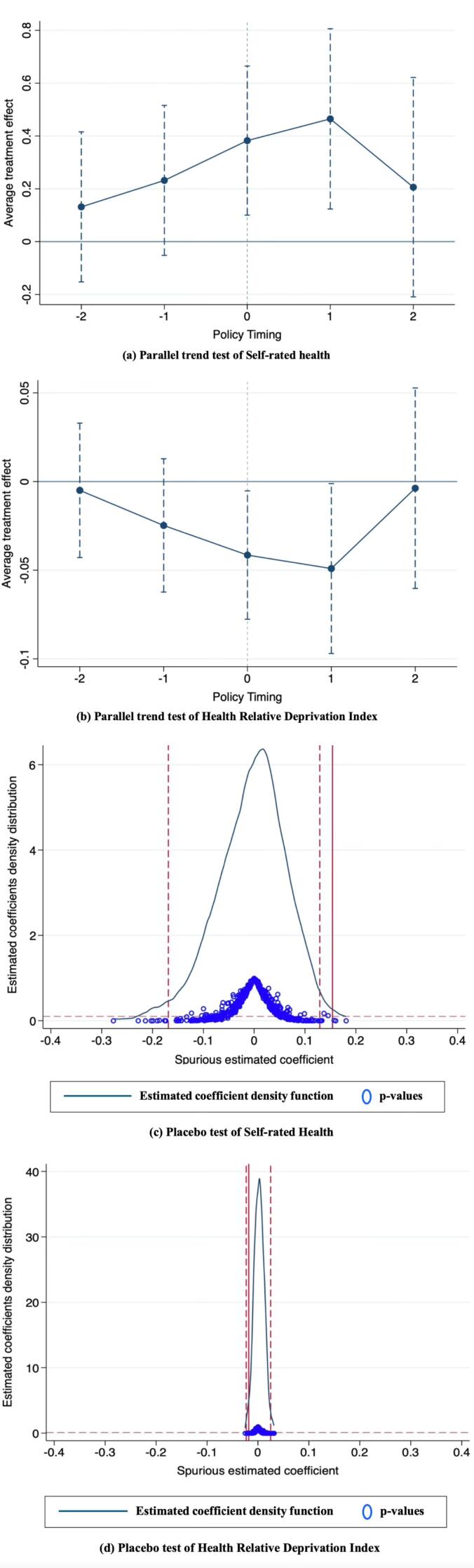
Results of the Robustness Test. Note: In parts (c) and (d) of the figure, the dashed lines are the 2.5th and 97.5th percentile values (other than NRCMS-PPP), the solid line is the NRCMS-PPP value. Abbreviation: NRCMS-PPP, New Rural Cooperative Medical Scheme Provincial Pooling Policy.

 In empirical studies utilizing the DID model, it is essential to satisfy the parallel trends assumption. Given that, this study employs time-varying DID model, where different experimental groups experience policy shocks at different time points, the study utilizes an event analysis method to conduct dynamic effect tests. The results are shown as (a) and (b) in Figure 2. The vertical axis represents the average treatment effect of the provincial-level pooling policy. The results showed that there was no significant difference in SH and HRDI between the treated and control groups before the implementation of NRCMS-PPP. This indicates that the parallel trends assumption is satisfied.

####  Propensity Score Matching Difference-in-Differences 

 To mitigate selection bias, this study conducted a robustness check on the baseline regression results using the nearest neighbor matching method. The propensity score matching (PSM) method can alleviate selection bias by controlling for observable variables in the sample.^38^ After nearest-neighbor matching, the results of the balance test showed that there was no significant difference in any of the control variables between the treatment group and the control group, and the bias (%) in the standardized means of the control variables between the two groups was less than 10%, which was significantly smaller than that before pairing, indicating that the pairing effect was good. After PSM, the estimated coefficients on SH and HRDI among older population for the NRCMS-PPP are 0.156 (*P <* .01) and -0.019 (*P* = .01). This is generally consistent with the results of the benchmark regression, indicating that the previous regression results are robust and reliable. Please see appendix for specific results.

####  The Placebo Tests

 Although this study has evaluated the health performance of the NRCMS-PPP through methods such as time-varying DID and PSM-DID, the possibility that the observed effects were influenced by other random factors or policies cannot be completely ruled out. Therefore, further placebo tests are needed to validate the reasonability of the results. This study adopts a two-way randomized placebo test, simultaneously randomizing policy time and treatment group samples. After excluding samples from Hainan, Chongqing, Tianjin, Shanghai, and Beijing, five reform provinces are randomly generated, and the reform years are randomly assigned among remaining 18 provinces to construct pseudo-experiments. To improve the validity of the placebo test, this study repeated the regression 500 times. If the peak of the density distribution of the estimated coefficients from the placebo test deviates from the value of 0, it indicates the presence of chance factors or other policy influences, the reverse is not true. Parts (c) and (d) of Figure 2 show that the estimated coefficients of both SH and HRDI are clustered around 0, suggesting that the previous findings are robust.

###  Intermediary Mechanism

 Theoretically, the NRCMS-PPP will improve health and health inequalities in three ways. First, it facilitates cross-location medical care and enhancing the accessibility of medical service utilization.^23^ Second, it improves the level of medical protection in some areas with a lower level of economic development or where there is a gap in the health insurance fund.^24^ Third, it relaxes the regulation of health insurance funds, inducing moral hazard and leading to unnecessary medical treatment.^39^ For facilitating cross-location medical care, there is no appropriate variable to measure in the CLHLS. For the level of healthcare coverage and healthcare utilization, the total out-of-pocket medical expenses (TOME) and catastrophic health expenditure (CHE) were chosen to be measured in this study. The mediation effect test procedure is sequentially divided into three steps: firstly, test the effect of the NRCMS-PPP on older people’s SH and HRDI, which requires that the coefficient *β*_1_ is significant before the next test can be conducted, as has been verified above. Secondly, test the effect of the NRCMS-PPP on the mediator variables, and stop the test if the coefficient *η*_1_ is not significant. Finally, test the effect of the NRCMS-PPP and the mediator variables on the SH and HRDI of the older population. In models (5) and (6), *M*_iyp_ is the mediating variable, which includes the variables of TOME and CHE. For the TOME, the actual out-of-pocket total medical expenses of the sample in the past year are logarithmically treated. For the CHE, it is judged according to whether the ratio of the sample’s actual out-of-pocket total medical cost expenditures in the past year to the annual household income exceeds a certain percentage, and this study follows the accepted 40% as the cut-off,^40^ if it is greater than 40%, then suffering from major disease expenditure shocks is defined as 1, otherwise it is defined as 0.


(4) $$Y_{iyp}=\beta_{0}+\beta_{1}Policy_{iyp}+\delta X_{iyp}+\gamma_{i}+\mu_{y}+\delta_{p}+\varepsilon_{iyp}$$

(5) $$M_{iyp}=\eta_{0}+\eta_{1}Policy_{iyp}+\delta X_{iyp}+\gamma_{i}+\mu_{t}+\delta_{p}+\varepsilon_{iyp}$$

(6) $$Y_{iyp}=\lambda_{0}+\lambda_{1}Policy_{iyp}+\lambda_{2}M_{iyp}+\delta X_{iyp}+\gamma_{i}+\mu_{t}+\delta_{p}+\varepsilon_{iyp}$$

In Table 4, the second step shows that the NRCMS-PPP significantly reduces the probability of CHE among the older adults (estimated coefficients = -0.045; *P* = .054). The NRCMS-PPP significantly reduces the TOME of the older adults (estimated coefficients = -0.767; *P* <.01), which reduces the burden of medical expenditures of the older adults. Therefore, the significantly affected CHE and TOME are taken as the mediating variables for the next test.

**Table 4:** Results of the Mediation Effect Test of the NRCMS-PPP

**Second Step**				
**Variables**	**CHE**		**TOME**	
	** (1)**		** (2)**	
Provincial pooling	-0.045^*^ (-1.925)		-0.767^***^ (-3.919)	
Control variables	√		√	
Constant	1.146^***^ (3.136)		2.875 (1.025)	
Ind, Year, Prov	√		√	
Sample size	13217		13234	
R^2^	0.239		0.173	
**Third Step**				
**Variables**	**SH**		**HRDI**	
	** (1)**	** (2)**	** (3)**	** (4)**
Provincial pooling	0.136^***^ (2.504)	0.121^**^ (2.219)	-0.015^*^ (-1.780)	-0.013 (-1.521)
CHE	-0.245^***^ (-7.613)		0.038^***^ (7.838)	
TOME		-0.035^***^ (-10.839)		0.005^***^ (11.697)
Control variables	√	√	√	√
Constant	2.390^***^ (3.707)	2.190^***^ (3.312)	0.248^***^ (2.580)	0.281^***^ (2.853)
Ind, Year, Prov	√	√	√	√
Sample size	13217	13234	13217	13234
R^2^	0.254	0.258	0.258	0.262

Abbreviations: SH, self-rated health; HRDI, health relative deprivation index; NRCMS-PPP, New Rural Cooperative Medical Scheme Provincial Pooling Policy; TOME, total out-of-pocket medical expenses; CHE, catastrophic health expenditure. ***, ** and * represent significance at the 1%, 5%, and 10% level.

 The third step shows that after adding CHE to the baseline regression, the estimated coefficients of SH are significantly positive and those of CHE are significantly negative; the estimated coefficients of HRDI are significantly negative and those of CHE are significantly positive. This suggests that NRCMS-PPP plays a partial mediating effect through CHE, reducing the probability of “poverty caused by illness and returning to poverty due to illness” among the older population, thus improving their health status and reducing health inequality.

 After adding TOME to the baseline regression, the estimated coefficients for SH are significantly positive and those for TOME are significantly negative, while the estimated coefficients for HRDI are no longer significant and those for TOME are significantly positive. This suggests that on the SH side, NRCMS-PPP exerts a partial mediating effect through TOME. In the case of HRDI, NRCMS-PPP exerted a fully mediated effect through TOME. The NRCMS-PPP reduces the TOME of older adults and reduces their disease cost burden, thereby improving their health and reducing health inequities.

## Discussion

###  NRCMS-PPP Improved Health and Health Inequalities of Older Population

 The results of the study show that the NRCMS-PPP improved the SH of the older population and reduced their HRDI. These findings are consistent with previous studies^24,25^ By enhancing the fairness of the NRCMS system, improving the co-payment capacity and risk-resistance of the NRCMS fund, enabling participating farmers across the province to enjoy the same hospitalization reimbursement, and facilitating cross-location medical treatment, the NRCMS-PPP can help to increase the utilization of access to healthcare services by participants whose health is in a disadvantageous position, and thus effectively improve their health status and reduce their HRDI.^21^ However, there are also negative effects that may result from an increase in the level of pooling of the NRCMS. NRCMS-PPP improves the level of healthcare coverage and reduces the burden of disease costs on patients, but it is also prone to moral hazard problems. After the NRCMS-PPP, the lower-level health insurance management organizations have shifted their responsibilities upwards, and the lack of supervisory motivation may induce unnecessary medical behaviors of the insured.^26^ This unnecessary medical behavior has greatly increased medical expenses and reduced the efficiency of the health insurance fund, which is not conducive to the sustainable development of the health insurance fund.^39^ Therefore, in the process of raising the level of health insurance pooling, it is also necessary to strengthen the supervision of the health insurance fund, do a good job of hierarchical diagnosis and treatment, and guide patients to seek reasonable medical treatment. In addition, UREM is more effective than RTFM in promoting health, possibly because it makes better use of the “law of large numbers” of the health insurance fund, which allows for better fund mobilization and resource allocation.^24^ It should be noted that UREM is suitable for regions with a high level of economic development and small differences in economic levels within the region. For regions with a lower level of economic development or greater intra-regional differences in economic levels, the RTFM can be selected first, and then gradually transitioned to the UREM as the level of economic development and inter-regional differences are reduced.

 Moreover, this study did not run an additional placebo analysis using individuals who are not signed up for NRCMS. The reason behind this is that the individuals not signed up for NRCMS are a sample of those enrolled in public healthcare, Basic Medical Insurance for (Non-working) Urban Residents, the BMIURR, and the Basic Medical Insurance for Urban Workers. Their level of healthcare coverage is much larger than that of the NRCMS and is not comparable to the NRCMS, and the very small sample that is not enrolled in any insurance has no association with the NRCMS-PPP, and they are not good references for evaluating policy outcomes. Indeed, the placebo test in this study, which randomized changes in the timing of policy implementation and the provinces in which it was implemented, was highly reliable.

###  Heterogeneity Analysis of the Impact of the NRCMS-PPP on Self-Rated Health and Health Relative Deprivation Index 

 Analyzed from the educational status, the NRCMS-PPP is more conducive to improving the health of the older adults with the level of primary education, but more conducive to mitigating the health inequalities of the illiterate older adults. Generally, compared with the older adults with higher education, the older adults illiterate or only with primary school education have fewer social resources, lower income levels, and are in a disadvantaged position when accessing healthcare resources. The NRCMS-PPP helps to improve the health welfare level of the older adults illiterate or only with primary school education, thereby effectively improving their health status and promoting health equity.^33^

 From the point of view of place of residence, the NRCMS-PPP has helped to improve the health status of older population in rural areas and alleviate their health inequalities, but has not significantly improved the health and health inequalities of older population living in urban areas.^32^ This policy not only enhances the fairness of the NRCMS system, but also improves the co-payment capacity and risk-resistance of the NRCMS fund. By applying the same reimbursement rate for hospitalization to all participating farmers in the province, the policy helps to alleviate the contradictions between urban and rural areas in terms of unbalanced utilization of medical services, unequal benefits and health inequities. The policy has increased hospitalization reimbursement rates for NRCMS participants living in rural areas, thereby facilitating their healthcare utilization and ultimately effectively improving their health and alleviating their health inequalities.

###  The Mediating Effects of Catastrophic Health Expenditure and Total Out-of-Pocket Medical Expenses in NRCMS-PPP

 The NRCMS-PPP reduces the probability of the older adults experiencing CHE and reduces their burden of disease costs, thus improving their health and reducing health inequalities. Take Shanghai for example, after the implementation of the NRCMS-PPP, the proportion of compensation within the scope of policy for community health service centers, secondary and tertiary medical institutions is 80%, 75% and 50%, respectively, with an average of 75%. The ceiling for inpatient and outpatient major disease compensation is 120 000 yuan, and if the accumulated out-of-pocket expenses within the scope of the policy for that year still exceed 10 000 yuan after being compensated by the medical fund of the NRCMS, the exceeding portion of the out-of-pocket expenses will then be paid at a further 70%, and the ceiling will be 80 000 yuan. It can be seen that NRCMS-PPP effectively reduces the burden of patients’ disease costs, helps farmers to seek timely medical treatment when they suffer from serious illnesses, and reduces the phenomenon of “poverty due to illness and return to poverty due to illness.”^33,39^ However, it is also necessary to strengthen the regulation of medical insurance to prevent excessive medical treatment.

###  Limitations

 Due to the limitations of the publicly available database, Ningxia, Qinghai, and Tibet were not included in the study among all eight provinces in China that have implemented the NRCMS-PPP. Only 5 provincial-level regions were included in the experimental group scope in this study, including Beijing, Tianjin, Shanghai, and Chongqing, which are municipalities directly under the central government; and Hainan Province, which is on an island with significant differences from normal provinces. There are certain limitations in terms of representativeness and experience promotion of the 5 provincial-level regions for other provinces. In addition, the SH indicators and HRDI were used in this study to reflect individual health status and health inequalities. However, these indicators are subjective. Future research should focus on enriching and refining indicators of health and health inequalities.

## Conclusion

 The NRCMS-PPP reduces the probability of the older adults experiencing CHE and reduces their burden of disease costs, thus improving their health and reducing their health inequality. Policy effects vary in terms of educational status and areas of residence. The NRCMS-PPP helps participants from health disadvantaged groups to access and utilize medical services by increasing the fairness of the medical care system and facilitating access to medical care in other places, thereby improving their health and reducing their HRDI.

## Ethical issues

 The study complies with the current laws of the country in which it was performed. The CLHLS is publicly available, and all procedures involving research study participants were approved by the biomedical ethics committee of Peking University (IRB00001052–24713074). The consent was obtained from all of the participants.

## Conflicts of interest

 Authors declare that they have no conflicts of interest.

## Data availability statement

 The datasets generated and analyzed during the current study are available in the CLHLS repository [https://opendata.pku.edu.cn/dataverse/CHADS].

## Endnotes


^[1]^ For missing values of control variables such as marriage, education, and smoking for an individual in the database, this study checked and added them based on the marital, educational, and smoking histories completed by the individual in other periods of time.
^[2]^ A total of 21 Beijing samples were removed. The coefficient for SH before removal was 0.149, *P* <.01; the coefficient for HRDI was -0.018, *P* = .02.
